# Integrative Survey of 68 Non-overlapping Upstate New York Watersheds Reveals Stream Features Associated With Aquatic Fecal Contamination

**DOI:** 10.3389/fmicb.2021.684533

**Published:** 2021-08-12

**Authors:** Hyatt Green, Maxwell Wilder, Martin Wiedmann, Daniel Weller

**Affiliations:** ^1^Department of Environmental Biology, College of Environmental Science and Forestry, State University of New York, Syracuse, NY, United States; ^2^Department of Food Science, Cornell University, Ithaca, NY, United States

**Keywords:** microbial water quality, *Escherichia coli*, land use, Finger Lakes, microbial source-tracking

## Abstract

Aquatic fecal contamination poses human health risks by introducing pathogens in water that may be used for recreation, consumption, or agriculture. Identifying fecal contaminant sources, as well as the factors that affect their transport, storage, and decay, is essential for protecting human health. However, identifying these factors is often difficult when using fecal indicator bacteria (FIB) because FIB levels in surface water are often the product of multiple contaminant sources. In contrast, microbial source-tracking (MST) techniques allow not only the identification of predominant contaminant sources but also the quantification of factors affecting the transport, storage, and decay of fecal contaminants from specific hosts. We visited 68 streams in the Finger Lakes region of Upstate New York, United States, between April and October 2018 and collected water quality data (i.e., *Escherichia coli*, MST markers, and physical–chemical parameters) and weather and land-use data, as well as data on other stream features (e.g., stream bed composition), to identify factors that were associated with fecal contamination at a regional scale. We then applied both generalized linear mixed models and conditional inference trees to identify factors and combinations of factors that were significantly associated with human and ruminant fecal contamination. We found that human contaminants were more likely to be identified when the developed area within the 60 m stream buffer exceeded 3.4%, the total developed area in the watershed exceeded 41%, or if stormwater outfalls were present immediately upstream of the sampling site. When these features were not present, human MST markers were more likely to be found when rainfall during the preceding day exceeded 1.5 cm. The presence of upstream campgrounds was also significantly associated with human MST marker detection. In addition to rainfall and water quality parameters associated with rainfall (e.g., turbidity), the minimum distance to upstream cattle operations, the proportion of the 60 m buffer used for cropland, and the presence of submerged aquatic vegetation at the sampling site were all associated based on univariable regression with elevated levels of ruminant markers. The identification of specific features associated with host-specific fecal contaminants may support the development of broader recommendations or policies aimed at reducing levels of aquatic fecal contamination.

## Introduction

Aquatic fecal contamination poses risks to human and environmental health through the introduction of pathogens (Craun et al., 2006; Arnone and Walling, 2007), nutrients (Sharpley et al., 2003), antimicrobials (Chee-Sanford et al., 2009; Karkman et al., 2019), and hormones (Boxall et al., 2003; Hanselman et al., 2003; Combalbert and Hernandez-Raquet, 2010; Bartikova et al., 2016). From 1978 to 2014, contact with contaminated untreated recreational water (e.g., lakes) resulted in 184 outbreaks associated with acute gastrointestinal illness, acute respiratory illness, and skin-related illness among other symptoms (CDC, 2019). Food-borne outbreaks can also be caused by using contaminated water for various agricultural purposes, such as frost protection and irrigation during produce production (FDA, 2018). Harmful algal blooms that pose both human and environmental health risks are also caused by, at least in part, phosphorus-rich runoff from fertilizer or manure applied to agricultural fields (Kane et al., 2014; Vadas et al., 2017; Kumaragamage and Akinremi, 2018). Therefore, the identification of not only the origins of fecal contaminants but also the factors that affect their transport, storage, and decay is essential to protecting ambient water quality and human and environmental health.

The protection and remediation of surface water quality often begins with assessing potential hazards, including sources of fecal contamination (WHO, 2016). A multitude of factors that affect levels of observed aquatic fecal contamination have been identified. Examples of well-known factors include, but are not limited to, recent rainfall and stormwater runoff (VanWormer et al., 2016), livestock density in the upstream catchment (Oliver et al., 2018), and poorly maintained wastewater infrastructure (Ahmed et al., 2018). However, many sources are diffuse and/or intermittent and may be difficult to pinpoint. Previous studies have assessed the association between the presence and levels of fecal indicator bacteria (FIB), such as *Escherichia coli* or fecal coliforms, and various factors thought to affect their distribution. However, it is well-established that FIB are ubiquitous in mammalian hosts, including some wildlife, and represent the total level of fecal contamination in a water body (Dufour, 1984). Therefore, it is difficult to gage the importance of the different factors controlling the input of a single fecal type (e.g., human and ruminant) using FIB as a response because measured FIB levels are a composite of FIB from different sources that may have different origins, routes of introduction, and other factors that control their distribution.

Over the last two decades, microbial source-tracking (MST) methods that target host-specific bacteria (Bernhard and Field, 2000; Mieszkin et al., 2010; Green et al., 2012) or viruses (Zhang et al., 2005; Stachler et al., 2017) have been used to distinguish and quantify levels of source-specific fecal contaminants in water samples. MST has most often been used to facilitate remediation of specific waterbodies, but could be used as a model input to identify and rank the major factors controlling different types of fecal contaminants. Such information could inform site-specific, watershed-scale, or regional management plans to help avoid sourcing or using water for agriculture or recreation that presents a risk to human health, such as water contaminated with human sewage or septage.

In this study, we visited 68 streams over a single growing season in the Finger Lakes region of Upstate New York, United States, and collected weather, land-use patterns, and water quality data (e.g., *E*. *coli*, MST markers, and physical–chemical parameters), as well as other stream features with the goal of identifying factors that affect fecal contamination at a regional scale. Our specific objectives were to (i) identify the associations between levels of *E*. *coli*, the detection of MST markers (i.e., avian, canid, human, and ruminant), and the presence of potential sources of upstream fecal contamination, (ii) characterize the relationship between *E*. *coli* levels and MST marker detection, and (iii) identify key spatial, weather, and physical–chemical water quality factors associated with an increased or decreased likelihood of fecal contamination.

## Materials and Methods

### Spatial Analysis

#### Land Cover Characterization

Inverse-distance weights (IDW) were used to characterize land cover as described previously (Weller D.L. et al., 2020). Land cover percentages were calculated for the following distance intervals around each sampling site: 0–100, 100–250, 250–500, 500–1,000, 1,000–2,000, 2,000–5,000, 5,000–10,000, 10,000–20,000, and >20,000 m. The IDW proportion under each land cover class was then calculated for the total watershed and stream corridor (60 m buffer from the stream channel) using a modified version of the equation from King et al. (2005) (example R code available at https://github.com/wellerd2).

#### Feature Density

The presence, density, and flow path distance (minimum and median) from the sampling site to features that could affect measured water quality parameters were also determined. Flow path distances were estimated by creating flow networks using the National Hydrology Dataset (NHD) flow accumulation and flow direction rasters, as well as NHD flowline data, and analyzed using the riverdist package version 0.15.3 (Tyers, 2017) in R as done previously (Weller et al., 2019). Flowline data were used for features that intersected streams (e.g., roads), while flow networks were used for all other features. The only data available for septic systems were aggregated to census tracts; therefore, septic density in each watershed was interpolated based on the percentage overlap between each census tract and the watershed as described previously (Weller et al., 2019).

#### Sample Site Selection

Streams and possible access points were identified using ArcGIS 10.2. Stream access points that represented an upstream watershed area ≥10 km^2^ with streams adjacent (<400 m) to fields used to grow produce covered by the Food Safety Modernization Act over 4 of the past 8 years were identified as candidate sampling sites. From the candidate sampling sites, 68 sampling sites were randomly selected to ensure that the sampled watersheds did not overlap. While funding for this work was contingent on the confidentiality of precise sampling locations, all samples were collected near commercial farms from waterways used for irrigation in the Great Lakes or Finger Lakes watersheds. A map of approximate sampling locations is provided in a previous publication (Weller et al., 2019).

### Meteorological Data Acquisition

Meteorological data were obtained from the nearest Network for Environment and Weather Applications (NEWA) weather station.^1^ Total rainfall, average air temperature, and average solar radiation were then calculated using non-overlapping periods [i.e., 0–1, 1–2, 2–3, 3–4, 4–5, 5–10, 10–20, and 20–30 days before sample collection (d BSC)] accounting for the precise time of day the sample was collected.

### Sample Collection

Between April and October 2018, streams were sampled either two (*n* = 8 streams) or three (*n* = 60 streams) times each resulting in a total of 196 1-liter samples. Samples were stored on ice during transit and filtered within 6 h of sample collection. To create split samples for MST and enumeration of fecal indicators, 1-liter samples were shaken by hand before filtration of two separate 100 ml aliquots.

### Microbiological Analysis and MST

#### Enumeration of *E. coli*

*Escherichia coli* were enumerated using the Colilert Quanti-Tray 2000 kit (IDEXX, Westbrook, ME, United States) following the manufacturer’s instructions. Sample dilution was not performed so the upper and lower limits of quantification were 2,419.6 Most Probable Number (MPN)/100 ml and 1 MPN/100 ml.

#### Sample Processing for MST

One hundred milliliter subsamples were filtered using sterile, pre-bagged, single-use vacuum filtration units with 0.45 μm pore-size 47 mm diameter polyethersulfone filters. Filters were transferred to Lysing Matrix E tubes (MPBio, Irvine, CA, United States) and stored at −80°C for between 55 and 218 days (mean = 197 days) until DNA extraction. Prior to extraction, filters were allowed to thaw to room temperature, and a 29.2 μl aliquot of prepared *Caenorhabditis elegans* lysate was added to each tube as described previously (Kirtane et al., 2019). Following thawing and lysate addition, filters were homogenized on a FastPrep-24-5G (MPBio), and DNA was extracted with the DNeasy Blood and Tissue Kit (Qiagen, Hilden, Germany) with a final elution volume of 100 μl. Eluted DNA was stored at −20°C for between 1 and 27 days (mean = 21 days) until qPCR. Two DNA extraction blanks were included in each of the five extraction batches by conducting extractions as described above, but omitting the filters.

#### qPCR for MST

qPCR assays for human (HF183; Bernhard and Field, 2000; Green et al., 2014a), ruminant (Rum2Bac; Mieszkin et al., 2010), canine (DG3; Green et al., 2014b), avian (GFD; Green et al., 2012; Weller et al., 2019), and internal control *C. elegans* (CG4; Kirtane et al., 2019) molecular markers were performed as described previously (Green et al., 2019; Weller et al., 2019). Oligonucleotides were obtained from IDT (IA, United States), except for MGB probes that were obtained from Applied Biosystems (MA, United States). A version of the GFD assay that was modified for probe-based detection and that detects fecal contamination from gulls, geese, ducks, and chickens was used as previously reported (Weller et al., 2019). Gulls, geese, and ducks are common and are therefore likely sources of fecal contamination in the study area. Duplicate 25 μl qPCR reactions were prepared using 12.5 μl TaqMan Environmental Master Mix (ThermoFisher, Waltham, MA, United States), molecular grade water, primers and probes, and 2 μl DNA template. Reactions were run under default cycling parameters (50°C for 2 min; 95°C for 10 min; and 40 cycles of 95°C for 15 s, and 60°C for 1 min) on either a QuantStudio3 or QuantStudio5 (ThermoFisher). Each 96-well qPCR plate contained at least four no template control reactions (NTCs) for which molecular grade water was substituted in place of DNA template, as well as two wells of positive control consisting of 10^3^ copies/reaction of a custom designed gBlock (IDT). The automatic baseline setting and a threshold value of 0.03 were used to estimate C_*T*_ values. qPCR assays used in this study and their performance metrics are reported elsewhere (Weller et al., 2019).

#### qPCR Quality Control and Data Analysis

Assay-specific standard curves generated using 10^0^–10^6^ copies/reaction of custom-designed gBlocks (IDT) were used to convert C_*T*_ values to copy number per reaction. Reactions with C_*T*_ values greater than the intercept of the standard curve were considered below limits of detection (LOD). For each assay, a sample was considered detectable for MST markers only if both C_*T*_ values were within the LOD. Amplification was not detected in any of the NTC (*n* = 100) or extraction blank reactions (*n* = 20). Recovery of spiked *C. elegans* DNA estimated using the CG4 assay was used as a proxy for total DNA recovery through the extraction process as done previously (Kirtane et al., 2019). DNA recoveries ranged from unmeasurable due to no recovery of spike (one sample) to >100% indicating a large variability in recovery (Supplementary Figure 1). Because samples with low recoveries still provide informative data (e.g., detection of MST markers), no samples were removed from analysis based on their estimated recovery values. Kinetic outlier detection for all CG4 reactions was used to detect the possible effects of qPCR inhibition as reported previously (Bar et al., 2011; Green and Field, 2012; Kirtane et al., 2019). Briefly, a sigmoidal model was fit to each CG4 amplification curve from which the first and second derivative maxima were calculated. A difference of ≥10 fractional cycle values between the derivative maxima was considered a sign of significant inhibition in the reaction well. Using these methods, no samples displayed signs of qPCR inhibition; therefore, no samples were excluded from analysis because of qPCR inhibition.

### Data Analysis

#### Generalized Linear Mixed Models

General or generalized linear mixed models (GLMMs) were developed using the lme4 version 1.1 (Bates et al., 2015) and censReg version 0.5 (Henningsen, 2017) packages in R. Model outcomes were the presence or absence of the target MST marker or the log10 MPN of *E. coli*/100 ml. For models where the outcome was binary, a logit link was used. For models where the outcome was *E. coli* concentration (or “level”), censored regression was used to account for samples where the MPN was above the range of quantification (2,419.6 CFU/100 ml). No samples were below the limits of quantification. Week of the year was included as a fixed effect in all models to account for non-independence of samples due to the collection of samples in the same week. While stream ID was not itself a factor of interest, it was included as a random effect to account for pseudo-replication in all GLMMs.

#### Conditional CART Model Development

Conditional CART models [conditional inference trees (CTrees)] were included as an additional statistical technique that is robust to correlation and missingness and inherently considers hierarchical relationships within the data. CTrees were developed using the partykit package version 1.2 (Hothorn et al., 2006; Hothorn and Zeileis, 2015) using the following control parameters: mincriterion = 0.80, minbucket = 5, minsplit = 15, and maxsurrogate = 3. Separate models were developed for each outcome: (i) the presence or absence of the target MST marker or (ii) the log10 MPN of *E. coli*/100 ml. For models where *E. coli* levels were the outcome, the nine samples that were above the range of quantification maximum (2,419.6 CFU/100 ml) were assigned a value of 2,500 when developing the CTrees.

## Results

### FIB and MST Summary

*Escherichia coli* was detectable in all streams and samples with a mean value of 212 MPN/100 ml (Standard Deviation [SD] = 637 MPN/100 ml); nine samples were above the range of quantification. MST markers were less prevalent than *E. coli*, occurring in only 68% of streams and only 38% of samples. HF183 (a human fecal marker) was the most prevalent MST marker, followed by Rum2Bac (a ruminant fecal marker), GFD (an avian fecal marker), and DG3 (a canid fecal marker; Table 1). Rum2Bac marker concentrations were higher than HF183, GFD, and DG3. GFD and DG3 marker levels were approximately one and two orders of magnitude below HF183 and Rum2Bac marker levels, respectively. The scarcity of GFD (found in only 4% of samples) and DG3 (found in only 0.5% of samples) markers greatly limited our ability to draw conclusions about drivers of avian and canid contamination, respectively. GLMMs indicated that the probability of finding both human (4.520, *p* < 0.001, 95% CI = 2.012–10.155) and ruminant (6.838, *p* < 0.001, 95% CI = 2.406–19.433) markers increased with elevated *E. coli* levels.

**TABLE 1:** Study-wide detection frequency and concentrations of molecular markers.

	Number of positive samples (%)	Number of positive streams (%)	Geometric mean concentration in positive samples (copies/100 ml)	Median concentration in positive samples (copies/100 ml)	Minimum concentration in positive samples (copies/100 ml)	Maximum concentration in positive samples (copies/100 ml)
DG3	1 (0.5%)	1 (1%)	76	76	76	76
GFD	8 (4%)	7 (10%)	410.7	466.5	64	7,040
HF183	49 (25%)	31 (46%)	1,640.1	1,204	48	320,448
Rum2Bac	34 (17%)	26 (38%)	1,971.6	1,315.5	145	117,605

### Factors Associated With *E. coli* Concentration

#### GLMMs

Results from GLMMs indicated that the density of upstream pig farms had a significant positive effect on *E. coli* concentrations (1.868, *p* = 0.036, 95% CI = 0.121–3.615; Supplementary Table 1), while the presence of upstream stormwater outfalls was also associated with higher levels of *E. coli* (0.309, *p* = 0.008, 95% CI = 0.083–0.536). Interestingly, the presence of upstream goat/sheep farms had a significant negative effect on *E. coli* concentrations (−1.049, *p* = 0.018, 95% CI = −1.919 to −0.180). Despite being one of the most common forms of livestock in the study area, there was no clear association between dairy/cattle farm density and *E. coli* concentrations (−0.161, *p* = 0.071, 95% CI = −0.336 to 0.014). Increased dissolved oxygen (−0.155, *p* < 0.001, 95% CI = −0.204 to −0.106) and pH (−0.468, *p* < 0.001, 95% CI = −0.718 to −0.219) were associated with lower levels of *E. coli*, while turbidity was associated with higher levels (0.580, *p* < 0.001, 95% CI = 0.376–0.784). Average rainfall, temperature, and solar radiation also had a significant effect on *E. coli* levels. Total rainfall within 0–2 d BSC was associated with higher levels of *E. coli* (0–1 d BSC, 0.375, *p* < 0.001, 95% CI = 0.252–0.497; 1–2 d BSC, 0.194, *p* < 0.001, 95% CI = 0.099–0.289), while total rainfall 5–10 d BSC was somewhat negatively associated with *E. coli* levels (−0.077, *p* = 0.007, 95% CI = −0.132 to −0.021). Average temperature 5–10 days prior to sample collection was more strongly associated with *E. coli* levels (0.046, *p* < 0.001, 95% CI = 0.029–0.062) than average temperature 0–5 days prior to sample collection (0.019, *p* < 0.019, 95% CI = 0.003–0.035). Average solar radiation 4–5 days prior to sampling was positively associated with *E. coli* (0.455, *p* < 0.001, 95% CI = 0.189–0.721), while average solar radiation 0–2 days prior was negatively associated with *E. coli* (0–1 day prior, −0.387, *p* = 0.008, 95% CI = −0.674 to −0.101; 1–2 days prior, −0.399, *p* = 0.008, 95% CI = −0.696 to −0.103). The presence of exposed rock in the form of cobble, boulder, or bedrock in the streambed was associated with lower levels of *E. coli* (−0.434, *p* < 0.001, 95% CI = −0.641 to −0.228), while the presence of organic matter was significantly associated with higher levels of *E. coli* (0.440, *p* < 0.001, 95% CI = 0.225–0.656).

#### CTrees

**FIGURE 1:**
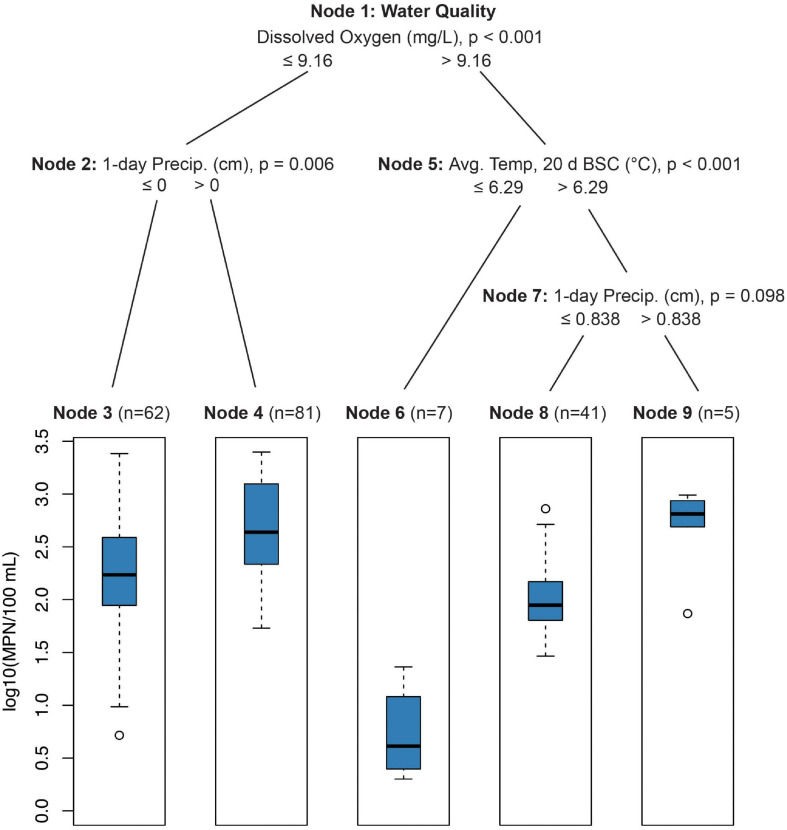
Conditional inference tree (CTree) showing factors and combinations of factors predictive of log_10_ *E. coli*/100 ml. In Node 1, dissolved oxygen was designated the primary split (*p* < 0.001), while pH (split = 8.26, *p* < 0.022) and turbidity (split = 0.50 log_10_ NTUs, *p* < 0.001) were designated surrogate splits that split the data equally well. Turbidity was the only significant surrogate split for Node 2 (split = 0.86 log_10_ NTUs, *p* < 0.007). For Node 5, average 10-day (split = 23.6°C, *p* < 0.003) and 30-day (split = 24.6°C, *p* < 0.002) temperatures were significant surrogates. Node 7 had no significant surrogates.

Conditional inference trees indicated that elevated *E. coli* levels were driven by physical–chemical water quality parameters (i.e., dissolved oxygen, turbidity, and pH) and precipitation 0–1 d BSC (Figure 1). *E. coli* levels were lowest (mean = 5.5 MPN/100 ml, SE = 12.5) when dissolved oxygen was above 9.16 mg/L and the average temperature 20–30 days before sampling was below 6.3°C (Figure 1, Node 6; *p* < 0.001). *E. coli* levels were highest (mean = 496.6 MPN/100 ml, SE = 89.9) when dissolved oxygen was less than 9.16 mg/L and total rainfall 0–1 d BSC was greater than zero centimeters (Figure 1, Node 2; *p* = 0.006).

### Factors Associated With Human MST Markers

#### GLMMs

Somewhat surprisingly, rural features, such as the presence of horse stables upstream of sampling points (3.409, *p* = 0.020, 95% CI = 1.216–9.556; Supplementary Table 2) and IDW % of pasture land use (0.968, *p* = 0.039, 95% CI = 0.938–0.998), were significantly associated with the presence of human markers. Also, as distance from upstream goat/sheep (1.065, *p* = 0.038, 95% CI = 1.003–1.131) or pig farms (1.084, *p* = 0.024, 95% CI = 1.011–1.163) increased, so did the probability of finding human markers, potentially reflecting rural to urban transitions. Unsurprisingly, the presence of upstream stormwater outfalls (5.297, *p* = 0.002, 95% CI = 1.879–14.929), wastewater discharges (4.426, *p* = 0.003, 95% CI = 1.672–11.722), and campgrounds (3.549, *p* = 0.024, 95% CI = 1.184–10.637) was associated with the presence of human markers. Total watershed area was also significantly associated with human marker presence (1.041, *p* = 0.006, 95% CI = 1.012–1.071). Rainfall 0–1 d BSC (3.412, *p* < 0.01, 95% CI = 1.729–6.732) and average solar radiation 2–3 d BSC (0.097, *p* = 0.008, 95% CI = 0.017–0.540) were also positively associated with human marker presence.

#### CTrees

**FIGURE 2:**
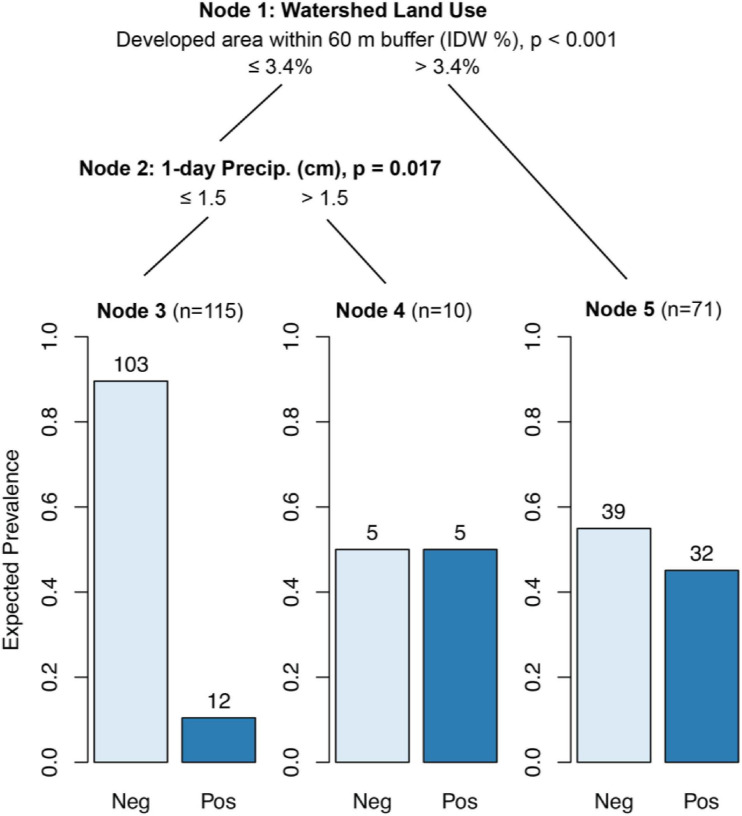
Conditional inference tree (CTree) showing factors and combinations of factors predictive of HF183 (human) marker presence. In Node 1, IDW % developed area within 60 m buffer was designated the primary split (*p* < 0.001), while developed watershed area (split = 41%, *p* < 0.002) and the presence of stormwater outfalls (*p* < 0.012) were designated surrogate splits that split the data equally well. Node 2 had no significant surrogates.

In areas where either the IDW developed area within a 60 m stream buffer exceeded 3.4%, the IDW developed watershed area exceeded 40.9%, or where there were stormwater outfalls present upstream, 45% of samples contained detectable levels of human markers (*p* < 0.05; Figure 2, Node 5). The probability of detecting human markers was lowest (13.6%) when none of the above conditions were met and rainfall 0–1 d BSC was less than 1.5 cm (*p* < 0.05; Figure 2, Node 3).

### Factors Associated With Ruminant MST Markers

#### GLMMs

The minimum distance to upstream cattle operations (0.842, *p* = 0.047, 95% CI = 0.710–0.997; Supplementary Table 3), IDW % of cropland within the 60 m buffer (1.054, *p* = 0.019, 95% CI = 1.009–1.101), and IDW % of forest/wetland (1.040, *p* = 0.036, 95% CI = 1.003–1.078) were all significantly associated with the presence of ruminant markers. Water quality parameters typically associated with poor water quality, such as high levels of *E. coli* (6.838, *p* < 0.01, 95% CI = 2.406–19.433) and turbidity (9.354, *p* < 0.01, 95% CI = 2.684–32.603), were also associated with the detection of ruminant markers as were average solar radiation 0–2 d BSC (0–1 d BSC, 0.041, *p* = 0.001, 95% CI = 0.007–0.261; 1–2 d BSC, 0.080, *p* = 0.005, 95% CI = 0.014–0.466) and total rainfall 0–1 d BSC (4.387, *p* < 0.01, 95% CI = 2.140–8.991) and 3–4 d BSC (2.770, *p* = 0.025, 95% CI = 1.139–6.734). Interestingly, the presence of exposed rock (0.257, *p* = 0.005, 95% CI = 0.099–0.669) and submerged aquatic vegetation (SAV) were also significantly associated with the presence of ruminant markers (0.278, *p* = 0.030, 95% CI = 0.087–0.885).

#### CTrees

**FIGURE 3:**
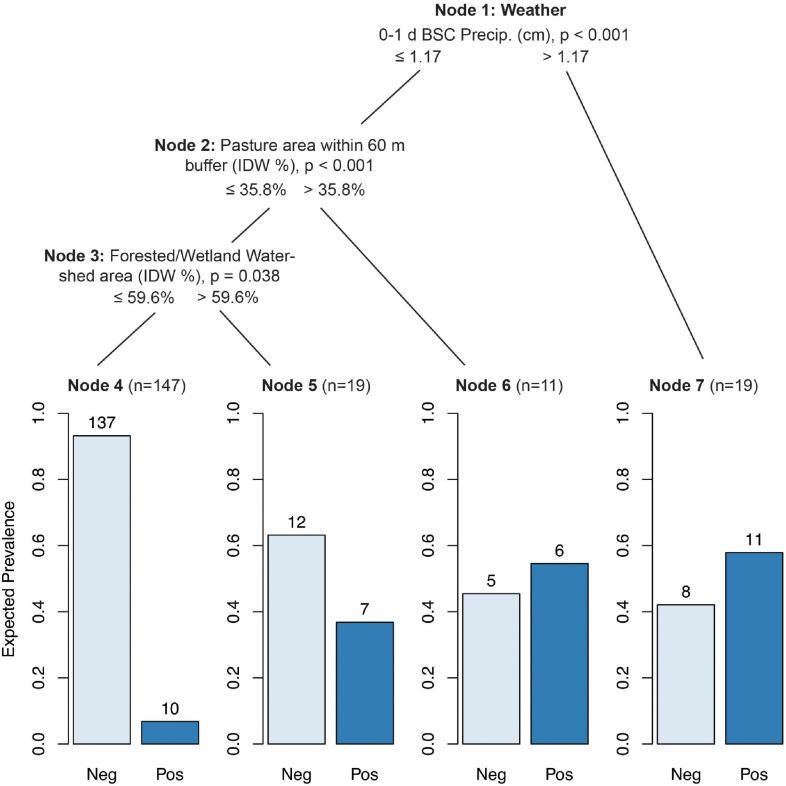
Conditional inference tree (CTree) showing factors and combinations of factors predictive of Rum2Bac (ruminant) marker presence. In Node 1, 0–1 d BSC precipitation was designated the primary split (*p* < 0.001), while *E. coli* (split = 2.8 log_10_ MPN/100 ml, *p* < 0.008), average solar radiation in the 0–1 d BSC (split = 0.43 MJ/m^2^, *p* < 0.008), and turbidity (split = 1.30 log_10_ NTUs, *p* < 0.056) were designated surrogate splits that split the data equally well. Nodes 2 and 3 had no significant surrogates.

While CTree results for ruminant marker presence/absence were more complex, they largely matched the results from the GLMMs. Ruminant markers were most likely to be found in samples with 0–1 d BSC precipitation greater than 1.17 cm (*p* < 0.001), *E. coli* levels greater than 631 MPN/100 ml (*p* = 0.008), average solar radiation greater than 0.43 MJ/m^2^ 0–1 d BSC (*p* = 0.008), or turbidity greater than 19.95 NTUs (*p* = 0.056; Figure 3, Node 7). When none of these conditions were met, ruminant markers were still more likely to be found if the pasture area within the 60 m buffer exceeded 35.8% (*p* < 0.001; Figure 3, Node 6). IDW % forested/wetland watershed area greater than 59.6% increased the probability of ruminant marker detection from 6.8% (Figure 3, Node 4) to 36.8% (Figure 3, Node 5; *p* = 0.038).

## Discussion

In this study, we used data collected from 68 streams to identify factors associated with fecal contamination across the Finger Lakes region of Upstate New York. Few studies have sampled this many streams multiple times to assess the causes of fecal contamination. While location-specific regulatory tools, such as total maximum daily loads (TMDLs) (U.S. Environmental Protection Agency, 1991), may offer pathways toward improved water quality for specific streams, they take years to develop and implement, and it is often resource-intensive to ensure compliance. On the other hand, federal standards for assessing human health hazard presence in recreational water (US EPA, 2012) and surface water used for produce production (Food and Drug Administration, 2015) may not fully account for spatiotemporal variation in microbial water quality, or the heterogeneity inherent to freshwater environments at local and regional scales (Truitt et al., 2018; Weller D. et al., 2020). In contrast, and intermediate in scale between the prior two approaches, the identification of factors associated with elevated levels of fecal contamination across a region may indicate effective mitigation strategies with relatively low overhead (Grayson et al., 1997; Verhougstraete et al., 2015). Although there were practical limitations that prevented an assessment of year-to-year variability on each of the 68 streams, follow-up studies should be conducted that assess this variability over a smaller number of streams.

*Escherichia coli* is an FIB and the target for existing agricultural and many recreational water quality standards and monitoring programs. In the present study, we primarily found associations between *E. coli* levels and meteorological or physical–chemical variables, although two land-use factors, upstream pig farm density and stormwater outfall presence, were also associated with elevated *E. coli* levels. More investigation is needed to determine why *E. coli* levels were negatively associated with goat/sheep farm density. It may be that goat/sheep farms may contribute lower levels of *E. coli* than larger livestock with higher amounts of waste, or that goat/sheep farms are associated with unmeasured factors that are also associated with low levels of *E. coli*. Rainfall is often associated with poor water quality (Pandey et al., 2012; Francy et al., 2013; Stocker et al., 2016; Weller et al., 2019) due to its propensity to promote the transport of contaminants into streams *via* stormwater runoff. The observed negative association between rainfall 5–10 d BSC and *E. coli* levels could be attributable to the rainfall-mediated removal of *E. coli* 5–10 BSC followed by limited terrestrial loading and/or limited transport due to little or no rainfall in the 0–5 d BSC window. The extended persistence and even growth of *E. coli* in the environment has been associated with elevated temperature previously (Porter et al., 2019) that is further supported by our observation of significant associations between *E. coli* and average temperature 0–10 days prior to sample collection. Our observation that average solar radiation 4–10 days prior to sampling had a positive effect on *E. coli* levels could be explained by the strong correlation between sunlight and warmer temperatures. In contrast, our observation that higher levels of solar radiation 0–2 d BSC had a negative effect on *E. coli* could be due to the fact that UV light damages cells directly.

Human-derived aquatic fecal contamination is often considered more dangerous than other sources because of its association with a diverse range of human pathogens (Soller et al., 2014). Human sources are often associated with point sources, such as stormwater outfalls, and non-point sources, such as failing septic tanks, and can be detected using a wide range of source-tracking methods including bacterial, viral, and chemical methods. Based on the occurrence of HF183 markers, we found that agricultural (e.g., presence of upstream stables), engineered (e.g., stormwater outfalls), and residential features (e.g., presence of upstream campgrounds), as well as the amount of developed area in the watershed (IDW %) and in the 60 m stream buffer area, were highly associated with signs of human fecal contamination. While the associations of HF183 markers with upstream campgrounds, stormwater outfalls, and wastewater discharges were not surprising, the associations of HF183 markers with upstream stable presence and the proportion of pasture land (IDW %) in the watershed were somewhat unexpected given that there is no clear source of human contamination from stables or pasture land. It is possible that stable presence may be associated with the presence of septic systems that could serve as inputs, or that the HF183 assay cross-reacted with equine or other non-human fecal contaminants. Cross-reaction of other human MST markers, but not HF183, with equine contaminants has been observed previously (Feng et al., 2020). Further investigation of the sites in question is needed to fully explain these associations.

A number of factors associated with high levels of *E. coli* were also significantly associated with the presence of ruminant contaminants. The associations of Rum2Bac markers with recent rainfall (GLMMs, 0–1 and 3–4 d BSC; CTree, 0–1 d BSC) suggest that a major driver of transport of ruminant contaminants to streams in the study region is stormwater runoff. Based on CTree analysis, even when rainfall was low, the probability of finding ruminant markers increased with pasture area within the stream buffer or forested/wetland area in the watershed (IDW %). It is currently unclear if the associations between Rum2Bac markers and forests or wetlands (both land-use types were collapsed in this study) were due to bovine sources or to other ruminants (e.g., deer) that can also be detected by the Rum2Bac assay (Mieszkin et al., 2010).

The observation that different factors are associated with *E. coli* than human markers could not only be due to the fact that *E. coli* is a composite measure of all fecal inputs, but also could be due to the extended persistence or resilience of molecular markers compared with cultivable indicators (Green et al., 2011). For example, the presence of upstream wastewater discharges was significantly associated with human markers but not *E. coli* levels. We attribute this to the observation that wastewater treatment processes are usually much more effective at reducing levels of cultivable *E. coli* than they are at reducing concentrations of DNA-based molecular markers (Wu et al., 2020), which are relatively stable. A similar situation may have occurred downstream of campgrounds where molecular markers may have persisted through the duration of transport from, presumably, septage, whereas the rapid decay of *E. coli* may have limited its detection in these areas. Human molecular markers may not be good indicators of some pathogens in scenarios where contaminant introduction to the waterbody is preceded by treatment or storage processes.

## Conclusion

The identification of a wide range of factors significantly associated with fecal contamination in the Finger Lakes region of Upstate New York streams points to the complex dynamics of fecal loading of streams in this area. Our observation that the presence of human contamination may be driven in some cases by watershed land use and specific features, such as stormwater outfalls, more so than meteorological factors suggests that limiting human fecal contamination of streams may be best confronted by management actions that prioritize spatial aspects of watersheds versus stream monitoring. However, the opposite may be true for mitigating ruminant contamination that appears to be highly associated with factors that are frequently monitored for in-stream (e.g., *E. coli*, turbidity, conductivity, temperature, and pH). Although the precise factors that control fecal contamination vary within and between watersheds, the factors identified herein could be useful for informing regional best management practices for reducing fecal contamination of waterbodies.

## Data Availability Statement

The raw data supporting the conclusions of this article will be made available by the authors without undue reservation.

## Author Contributions

MWe obtained funding for the collection of samples. HG obtained funding for the microbial source-tracking analysis and wrote the first draft of the manuscript and all authors edited subsequent drafts. MWe and DW conceived and designed the study. DW coordinated the collection of samples, water quality data, and spatial data including land use features and, conducted the statistical analysis. MWl collected the microbial source-tracking data. All authors approved the submitted version of the manuscript.

## Author Disclaimer

The content is solely the responsibility of the authors and does not represent the official views of the NIH or any other government agency.

## Conflict of Interest

The authors declare that the research was conducted in the absence of any commercial or financial relationships that could be construed as a potential conflict of interest.

## Publisher’s Note

All claims expressed in this article are solely those of the authors and do not necessarily represent those of their affiliated organizations, or those of the publisher, the editors and the reviewers. Any product that may be evaluated in this article, or claim that may be made by its manufacturer, is not guaranteed or endorsed by the publisher.

## Footnotes

1
newa.cornell.edu

## Supplementary Material

Supplementary Figure 1 Histogram of DNA recoveries (%) estimated by quantifying spiked *C. elegans*.

Supplementary Table 1 Parameters significantly associated with log-transformed *E. coli* concentrations using univariate GLMMs.

Supplementary Table 2 Parameters significantly associated with log-transformed HF183 marker presence/absence using univariate GLMMs.

Supplementary Table 3 Parameters significantly associated with log-transformed Rum2Bac marker presence/absence using univariate GLMMs.
