# Supplementary material for: Integrative Survey of 68 Non-overlapping Upstate New York Watersheds Reveals Stream Features Associated With Aquatic Fecal Contamination
Source: Front Microbiol. 2021 Aug 12;12:684533. doi: 10.3389/fmicb.2021.684533 (PMC8406625; doi:10.3389/fmicb.2021.684533)
Supplement: Supplementary Figure 1 — Histogram of DNA recoveries (%) estimated by quantifying spiked C. elegans. [file Data_Sheet_1.docx]

***Supplementary Tables***

Table S1. Parameters significantly associated with log-transformed E. coli concentrations using univariate GLMMs.

| **Variable Class** | **Variable** | **Estimate** | **p** | **Lower 95% CI** | **Upper 95% CI** |
| --- | --- | --- | --- | --- | --- |
| **Agricultural features** | |  |  |  |  |
|  | Density of upstream goat/sheep farms | -1.049 | 0.018 | -1.919 | -0.180 |
|  | Density of upstream pig farms | 1.868 | 0.036 | 0.121 | 3.615 |
| **Engineered features** | |  |  |  |  |
|  | Presence of stormwater outfall | 0.309 | 0.008 | 0.083 | 0.536 |
| **Water quality parameters** | |  |  |  |  |
|  | Dissolved oxygen | -0.155 | <0.001 | -0.204 | -0.106 |
|  | pH | -0.468 | <0.001 | -0.718 | -0.219 |
|  | log10 turbidity | 0.580 | <0.001 | 0.376 | 0.784 |
| **Land use** |  |  |  |  |  |
|  | IDW of forest-wetland prop. 60m | -0.007 | 0.045 | -0.014 | 0.000 |
| **Meteorological** | |  |  |  |  |
|  | Avg. solar radiation 0-1 d prior | -0.387 | 0.008 | -0.674 | -0.101 |
|  | Avg. solar radiation 1-2 d prior | -0.399 | 0.008 | -0.696 | -0.103 |
|  | Avg. solar radiation 4-5 d prior | 0.455 | 0.001 | 0.189 | 0.721 |
|  | Avg. solar radiation 5-10 d prior | 0.763 | <0.001 | 0.381 | 1.146 |
|  | Avg. temp 0-5 d BSC | 0.019 | 0.019 | 0.003 | 0.035 |
|  | Avg. temp 5-10 d BSC | 0.046 | <0.001 | 0.029 | 0.062 |
|  | Total rainfall 0-1 d BSC | 0.375 | <0.001 | 0.252 | 0.497 |
|  | Total rainfall 1-2 d BSC | 0.194 | <0.001 | 0.099 | 0.289 |
|  | Total rainfall 5-10 d BSC | -0.077 | 0.007 | -0.132 | -0.021 |
| **Streambed features** | |  |  |  |  |
|  | Presence of cobble, boulder or bedrock | -0.434 | <0.001 | -0.641 | -0.228 |
|  | Presence of org. matter | 0.440 | <0.001 | 0.225 | 0.656 |
|  |  |  |  |  |  |

Table S2. Parameters significantly associated with log-transformed HF183 marker presence/absence using univariate GLMMs.

| **Variable Class** | **Variable** | **Estimate** | **p** | **Lower 95% CI** | **Upper 95% CI** |
| --- | --- | --- | --- | --- | --- |
| **Agricultural features** | |  |  |  |  |
|  | Min. distance to upstream goat/sheep farms | 1.065 | 0.038 | 1.003 | 1.131 |
|  | Presence of upstream stables | 3.409 | 0.020 | 1.216 | 9.556 |
|  | Min. distance to upstream pig farms | 1.084 | 0.024 | 1.011 | 1.163 |
| **Engineered features** | |  |  |  |  |
|  | Presence of upstream stormwater outfalls | 5.297 | 0.002 | 1.879 | 14.929 |
|  | Presence of upstream WW discharges | 4.426 | 0.003 | 1.672 | 11.722 |
| **Geographic features** | |  |  |  |  |
|  | Watershed Area | 1.041 | 0.006 | 1.012 | 1.071 |
| **Water quality/Hydrologic parameters** | |  |  |  |  |
|  | log10 E. coli | 4.520 | <0.001 | 2.012 | 10.155 |
|  | Flow rate | 5.380 | 0.018 | 1.331 | 21.739 |
|  | log10 turbidity | 4.124 | 0.012 | 1.372 | 12.394 |
| **Land use** | |  |  |  |  |
|  | IDW of pasture prop. | 0.968 | 0.039 | 0.938 | 0.998 |
| **Meteorological features** | |  |  |  |  |
|  | Avg. solar radiation 2-3 d prior | 0.097 | 0.008 | 0.017 | 0.540 |
|  | Total rainfall 0-1 d BSC | 3.412 | <0.001 | 1.729 | 6.732 |
| **Residential features** | |  |  |  |  |
|  | Presence of upstream campgrounds | 3.549 | 0.024 | 1.184 | 10.637 |

Table S3. Parameters significantly associated with log-transformed Rum2Bac marker presence/absence using univariate GLMMs.

| **Variable Class** | **Variable** | **Estimate** | **p** | **Lower 95% CI** | **Upper 95% CI** |
| --- | --- | --- | --- | --- | --- |
| **Agricultural features** | |  |  |  |  |
|  | Min. distance to upstream cattle ops. | 0.842 | 0.047 | 0.710 | 0.997 |
| **Water quality parameters** | |  |  |  |  |
|  | log10 conductivity | 0.044 | 0.005 | 0.005 | 0.394 |
|  | log10 E. coli | 6.838 | <0.001 | 2.406 | 19.433 |
|  | pH | 0.056 | <0.001 | 0.013 | 0.248 |
|  | log10 turbidity | 9.354 | <0.001 | 2.684 | 32.603 |
|  | Water temperature | 0.905 | 0.045 | 0.821 | 0.998 |
| **Land use** | |  |  |  |  |
|  | IDW of cropland prop. 60m | 1.054 | 0.019 | 1.009 | 1.101 |
|  | IDW of forest-wetland prop. | 1.040 | 0.036 | 1.003 | 1.078 |
| **Meteorological** | |  |  |  |  |
|  | Avg. solar radiation 0-1 d prior | 0.041 | 0.001 | 0.007 | 0.261 |
|  | Avg. solar radiation 1-2 d prior | 0.080 | 0.005 | 0.014 | 0.466 |
|  | Total rainfall 0-1 d BSC | 4.387 | <0.001 | 2.140 | 8.991 |
|  | Total rainfall 3-4 d BSC | 2.770 | 0.025 | 1.139 | 6.734 |
| **Streambed features** | |  |  |  |  |
|  | Presence of cobble, boulder or bedrock | 0.257 | 0.005 | 0.099 | 0.669 |
|  | SAVs present or absent | 0.278 | 0.030 | 0.087 | 0.885 |

***Supplementary Figures***

**Figure S1.** Histogram of DNA recoveries (%) estimated by quantifying spiked *C. elegans*.


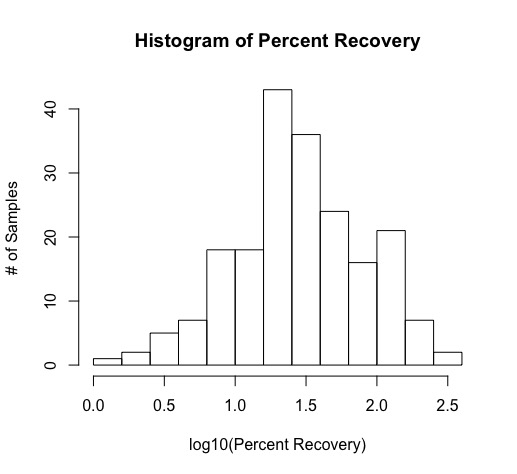


***Figure S1***
